# Treenuts and groundnuts in the EAT-Lancet reference diet: Concerns regarding sustainable water use

**DOI:** 10.1016/j.gfs.2020.100357

**Published:** 2020-03

**Authors:** Davy Vanham, Mesfin M. Mekonnen, Arjen Y. Hoekstra

**Affiliations:** a European Commission, Joint Research Centre (JRC), Ispra, Italy; b Robert B. Daugherty Water for Food Global Institute, University of Nebraska, Lincoln, NE, 68583, United States; c Twente Water Centre, University of Twente, P.O. Box 217, Enschede, Netherlands; d Institute of Water Policy, Lee Kuan Yew School of Public Policy, National University of Singapore, Singapore

**Keywords:** Water footprint, Diet, Nuts, Water, Food security, EAT-Lancet

## Abstract

The EAT-Lancet universal healthy reference diet recommends an increase in the consumption of healthy foods, among which treenuts and groundnuts. Both are, however, water-intensive products, with a large water footprint (WF) per unit of mass and protein and already today contribute to blue water stress in different parts of the world. The envisaged massive required increase in nut production to feed a global population with this reference diet, needs to occur in a water-sustainable way. In this paper, we identify and quantify where current nut production contributes to local blue water stress and discuss options for water-sustainable nut production. We show that 74% of irrigated nuts are produced under blue water stress (of which 63% under severe water stress), throughout many regions of the world, most notably in India, China, Pakistan, the Middle East, the Mediterranean region and the USA. We critically evaluate which nut types to promote given substantial differences in WFs. We propose sustainable intensification of nut production employing nut-specific WF benchmarks. We also recommend integrated water resources management including maximum sustainable levels of water consumption by setting of WF caps.

## Highlights

• The EAT-Lancet reference diet recommends increased nut consumption.
• Treenuts and groundnuts are water-intensive products.
• 74% of irrigated nuts are produced under blue water stress.
• 63% of irrigated nuts are produced under severe water stress.
• Increase in nut production needs to occur in a water-sustainable way.

## 1 Introduction

The proposed transformations in the food system including a universal diet described by Willett et al. (2019) include a significant increase in global consumption of treenuts and groundnuts. Both are nutrient-dense and contain primarily unsaturated fatty acids, fibre, vitamins, minerals, antioxidants, and phytosterols. They are also an important source of protein. Groundnuts (peanuts) have an average protein content of 257 g/kg, whereas the protein content of treenuts ranges from 18 g/kg (chestnuts) to 200 g/kg (almonds)(FAO, 2019)(SI Table 1). Treenuts and groundnuts are water intensive to produce, have large water footprints per unit of mass and protein (Mekonnen and Hoekstra, 2011) and contribute already today in different parts of the world to blue water stress (Fulton et al., 2019).

Willett et al. (2019) acknowledge that for dietary change, blue water use could increase by 1–9% as reductions related to lower consumption of animal products and sugar are overcompensated by increases related to greater consumption of nuts and legumes. They state that staying within the planetary boundary for water can be achieved by combining improvements in water-use efficiency with reductions in food loss and waste. However, they explicitly state that their analysis does not highlight regions or nations that currently face water shortage and are already above regional or national boundaries for environmental flow requirements. The regional aspect of blue water stress and the specific contribution of nut production is thus not addressed.

Therefore, in this perspective paper, we identify and quantify the blue and green water resources required for current treenut and groundnut production, where this production contributes to local blue water stress, and discuss options for water-sustainable nut production.

Crop production requires both blue and green water resources (Mekonnen and Hoekstra, 2011). Blue water refers to water in rivers, lakes and aquifers. Green water is the soil water held in the unsaturated zone, formed by precipitation and available to plants (Falkenmark et al., 2019). Irrigated agriculture receives blue water (from irrigation) as well as green water (from precipitation), while rain-fed agriculture receives only green water. Both resources are essential for food security (Hoekstra and Mekonnen, 2012; Vanham et al., 2018a), energy security (Mekonnen and Hoekstra, 2012; Vanham et al., 2019b), water security and the environment (Falkenmark et al., 2019). As both resources are essential for nut production, we discuss both.

The food system is a major cause of both blue water stress (WS)(Mekonnen and Hoekstra, 2016) and green water scarcity (Schyns et al., 2019). Within the SDG framework, blue WS is measured by means of SDG indicator 6.4.2 (Vanham et al., 2018b). The latter quantifies water abstraction related to environmentally available water resources, being total available water resources minus environmental flow requirements. Blue WS can however also be computed for consumptive water use. One of the most detailed geographical assessments has been done by Mekonnen and Hoekstra (2016). Also Willett et al. (2019) and Springmann et al. (2018) use consumptive water use to analyse how the food system can stay within the water planetary boundary. Here we analyse blue water stress of groundnuts and treenuts defined as consumptive water use related to environmentally available water resources. We do not discuss green water scarcity.

## 2 Methodology

First, we analyse the quantity of treenut and groundnut production for the current situation as well as the projected EAT-Lancet universal healthy reference diet scenario for the year 2050, based on FAOSTAT (2019) data and population projection data (UN, 2019). Second, we use the blue and green water footprint data of Mekonnen and Hoekstra (2011) to show the unit and total production water footprints of different treenut types and groundnuts. The water footprint (WF) quantifies both water consumption (blue plus green WF) and water pollution (grey WF)(Hoekstra and Mekonnen, 2012); we focus here on water consumption.

Third, we use the spatially distributed blue WS data of Mekonnen and Hoekstra (2016) and nut WF and irrigated yield data of Mekonnen and Hoekstra (2011) to assess where and in which quantity irrigated nut production contributes to and/or is produced under different levels of blue WS. We thereby put blue water footprint amounts (the accounting phase in a Water Footprint Assessment) in relationship to local blue water availability (the impact assessment phase) (Hoekstra et al., 2011).

Blue WS in Mekonnen and Hoekstra (2016) is computed as:

$$\text{Blue WS}=\frac{blue\;water\;footprint}{\left(WA-EFR\right)}$$

with WA = total water availability, and EFR = environmental flow requirements.

Following blue WS thresholds are used: values until 1 (low blue WS), 1–1.5 (moderate blue WS), 1.5–2 (significant blue WS) and more than 2 (severe blue WS). In the following discussion we only discuss blue WS as moderate, significant and severe blue WS. We do not include or discuss low blue WS. We assess groundnuts, cashew nuts, chestnuts, almonds, walnuts, pistachios and hazelnuts.

## 3 Results and discussion

### 3.1 Current and projected (EAT-Lancet universal healthy reference diet) treenuts and groundnut production

**Fig. 1:**
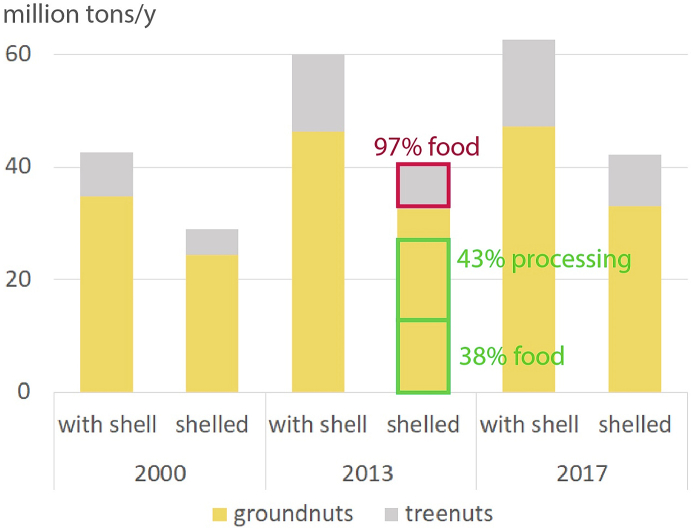
Production of groundnuts and treenuts in million tons/y.

Global treenut (shelled) production increased from 4.6 million tons in 2000 to 9.3 million tons in 2017, while global groundnut (shelled) production increased from 24.4 million tons to 33.0 million tons over the same period (FAOSTAT, 2019)(Fig. 1 and SI Table 2). In 2013, the most recent year in FAOSTAT that provides food balance data, of 32.5 million tons groundnuts produced were 38% used as direct food, 43% for processing (mainly oil) and the rest for other purposes. As for the 8.0 million tons (shelled) produced treenuts, 97% were used as direct food. In 2013, global average (direct food) consumption per capita equalled to 3.3 g of treenuts and 4.8 g of groundnuts per day. The reference diet recommends an average intake of 25 g of treenuts as well as 25 g of groundnuts per day. For a projected probabilistic median population of 9735 million people in 2050 (UN, 2019), this implies 89 million ton/y of shelled treenuts plus 89 million ton/y of shelled groundnuts (equivalent to 148 million ton/y of unshelled treenuts and 127 million ton/y of unshelled groundnuts, given a shell conversion fraction of 0.6 for treenuts and 0.7 for groundnuts (SI Table 2)). These rough estimates (that do not account for losses or waste along the supply chain and which assume the current production levels for processing of nuts and other purposes), require an increase in global nut production of more than 11 times current treenut production and 7 times current groundnut production.

### 3.2 Water resources for nut production

Over the period 1996–2005, the global average WF of treenut production has been estimated at 68,267 million m^3^/y (11,938 million m^3^/y blue, 56,329 million m^3^/y green), and the global average WF of groundnut production at 89,256 million m^3^/y (5107 million m^3^/y blue, 84,149 million m^3^/y green)(Mekonnen and Hoekstra, 2011)(SI Table 2). Among the treenuts, cashew nuts have the largest total WF (27,594 million m^3^/y, 1845 million m^3^/y blue, 25,749 million m^3^/y green). Groundnuts have the largest blue WF (5107 million m^3^/y), followed by pistachios (3507 million m^3^/y) and almonds (3013 million m^3^/y).

**Fig. 2:**
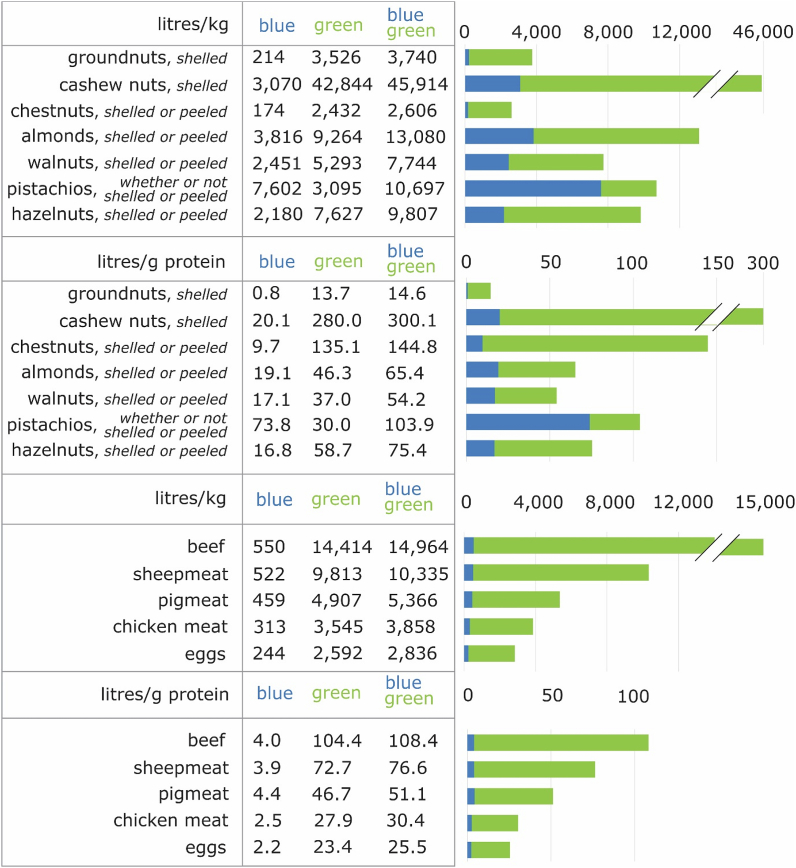
WF of different nut types (shelled) as well as some other food products for comparison, in litre/kg and litre per g of protein; the blue and green colours relate to blue respectively green water. Data source: Mekonnen and Hoekstra (2011) and Mekonnen and Hoekstra (2012). (For interpretation of the references to colour in this figure legend, the reader is referred to the Web version of this article.)

Large differences in total, blue and green average unit WFs exist between different nut types, both in terms of litre/kg and litre per gram of protein (Fig. 2). Cashew nuts have the largest average unit total WF (45,914 L/kg), followed by almonds (13,080 L/kg), pistachios (10,697 L/kg), hazelnuts (9807 L/kg) and walnuts (7744 L/kg). Groundnuts (3740 L/kg) and chestnuts (2606 L/kg) have considerable smaller total WFs per kg. Some nut types are on average primarily grown with green water, others with blue water. Regarding blue water, which is the focus of this paper, pistachios show the highest WFs per kg (7602 L/kg), followed by almonds (3816 L/kg), cashew nuts (3070 L/kg), walnuts (2451 L/kg) and hazelnuts (2180 L/kg). Groundnuts (214 L/kg) and chestnuts (174 L/kg) show considerable smaller WFs.

In terms of litre per gram of protein (Fig. 2), cashew nuts have the largest average unit total WF (300.1 L/g), followed by chestnuts (144.8 L/g), pistachios (103.9 L/g), hazelnuts (75.4 L/g), almonds (65.4 L/g) and walnuts (54.2 L/g). Groundnuts have considerable lower WFs (14.6 L/g). When only considering blue water, pistachios have by far the largest WFs (73.8 L/g). Relative similar values are observed for cashew nuts (20.1 L/g), almonds (19.1 L/g), walnuts (17.1 L/g) and hazelnuts (16.8 L/g). Chestnuts (9.7 L/g) and especially groundnuts (0.8 L/g) have considerable lower WFs.

In terms of litre per kg, almonds, pistachios, hazelnuts and walnuts have unit WFs in the range of red meat from ruminants, whereas groundnuts and chestnuts are in the range of white meat and eggs. In terms of litre per g protein, chestnuts and pistachios have unit WFs in the range of red meat from ruminants, whereas almonds, walnuts and hazelnuts are in the range of other red meat (pigs and sheep) and groundnuts lower than eggs.

### 3.3 Blue water stress (WS) related to nut production

**Fig. 3:**
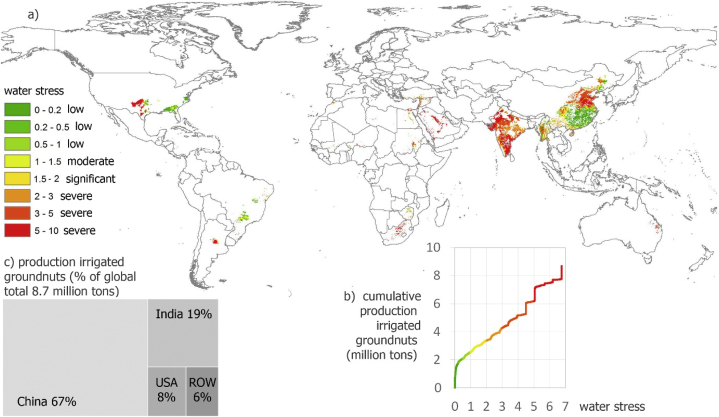
Production of irrigated groundnuts (with shell) under different levels of average annual water stress. Water stress occurs as from value 1 (a,b); c) production of irrigated groundnuts (with shell) according to main countries (ROW = Rest of world).

A significant proportion of irrigated groundnuts is produced under blue WS (values larger than 1, i.e. moderate to severe WS)(Fig. 3). Of a total annual production of 34.8 million tons (with shell), 8.7 million tons are irrigated. Of the latter, a total of 6.1 million tons contribute to and are produced under blue WS, of which 5.3 under severe blue WS, most of which in India and China. Other hotspot regions of irrigated groundnuts under WS are the USA and the Middle East.

**Fig. 4:**
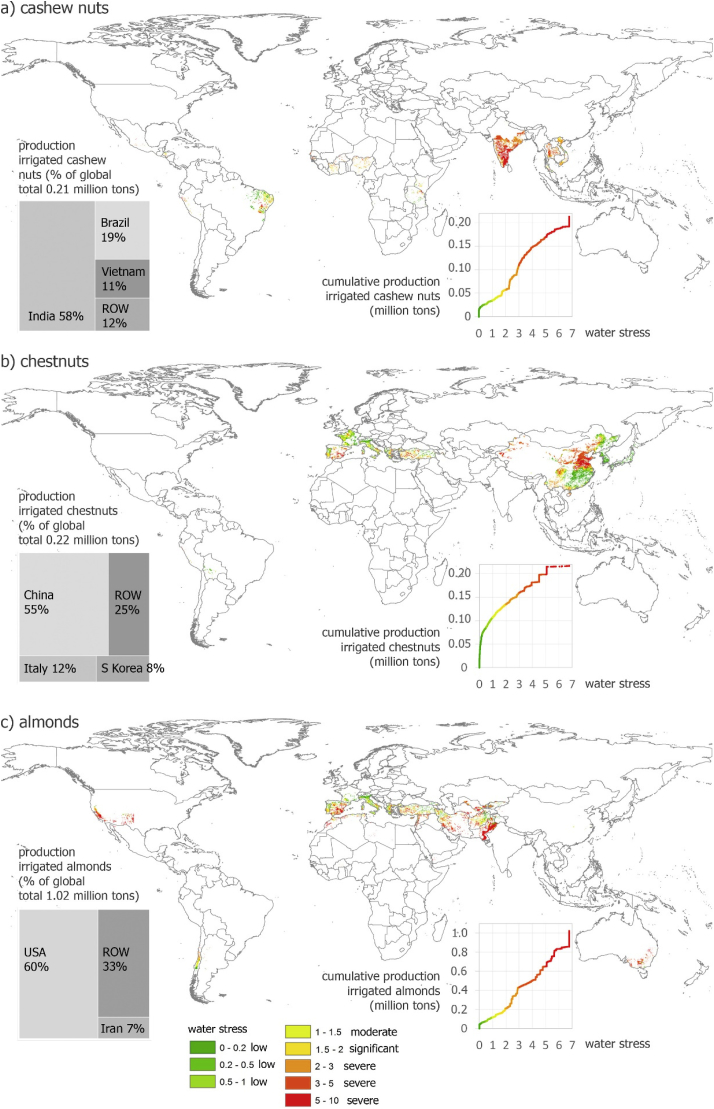
Production of irrigated cashew nuts (a), chestnuts (b) and almonds (c) under different levels of average annual water stress. Water stress occurs as from value 1. Indication of production of these irrigated nuts (with shell) according to main countries (ROW = Rest of world).

Irrigated production of cashew nuts and chestnuts amounts to each 0.2 million tons (with shell)(Fig. 4). For cashew nuts, 0.19 million tons are produced under blue WS, of which 0.17 million tons under severe WS, mostly in India, but also in Southeast Asia and Brazil. For chestnuts, 0.11 million tons are produced under blue WS, of which 0.08 million tons under severe blue WS, mostly in China and the Mediterranean region.

In total 0.9 million tons of 1.0 million tons irrigated almonds is produced under blue WS, of which 0.8 under severe blue WS (Fig. 4). The largest hotspot is California in the USA. But also in the Mediterranean region and the Middle East (such as Iran) large amounts of almonds are produced under blue WS.

**Fig. 5:**
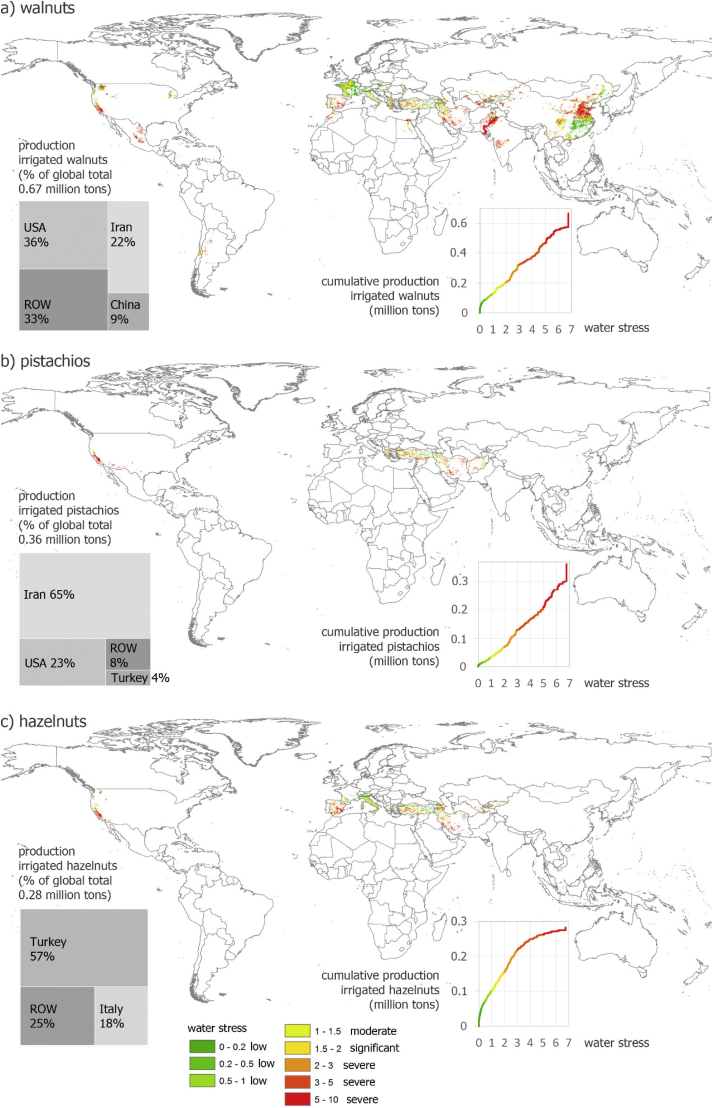
Production of irrigated walnuts (a), pistachios (b) and hazelnuts (c) under different levels of average annual water stress. Water stress occurs as from value 1. Indication of production of these irrigated nuts (with shell) according to main countries (ROW = Rest of world).

In total 0.59 million tons of irrigated walnut production (0.67 million tons) occurs under blue WS, of which 0.46 under severe blue WS (Fig. 5). Hotspots include the USA (California), Mexico, the Middle East (especially Iran), the Mediterranean (especially Turkey) and China.

Pistachios are primarily produced under blue WS in Iran, the USA (California) and Turkey (Fig. 5). Of a total irrigated production of 0.36 million tons, 0.33 are produced under blue WS, of which 0.30 under severe blue WS.

A large proportion of 0.18 million tons of 0.28 million tons irrigated hazelnuts is produced under blue WS, of which 0.13 under severe blue WS (Fig. 5). Hotspots are especially countries in the Mediterranean region such as Turkey and Italy, but also California in the USA.

These seven nut types account for in total 11.4 million tons of irrigated nuts, of which 8.4 million tons (74%) are produced under blue WS (moderate, significant or severe), and 7.3 million tons (63%) under severe blue WS.

### 3.4 Options for water-sustainable nut production

Willett et al. (2019) identify a range of strategies and solutions how to achieve a healthy diet within planetary boundaries. Many of them are applicable to nuts. According to Hoekstra (2014), there are three pillars for wise water use and allocation, which should ensure environmental sustainability, resource efficiency, and social equity. In the same order, these are, first, to implement water footprint caps for all river basins in the world (depending on water availability over time), second, the establishment of water footprint benchmarks for products (depending on the climate/soil-specific WFs associated with best practices) and third, fair water footprint shares per community, which includes the reconsideration of our consumption pattern. These measures are partly overlapping with the solutions and strategies presented by Willett et al. (2019). The first two define water-sustainable nut production.

In the line with these solutions, the most important options for water-sustainable nut production are displayed and discussed in Table 1.

**Table 1:** Options for sustainable nut production.

Option	Reasoning
Choice which nut type to produce	Due to substantial differences in average unit WF, both in terms of litre per kg and litre per gram of protein (Fig. 2). Groundnuts have generally smaller total and blue WFs both per kg and per gram of protein than tree nuts, but certain authors advocate to produce more perennial crops (treenuts) instead of annual crops (groundnuts) as they promise more sustainable agroecosystems (Crews et al., 2018).
Sustainable intensification of nut production to attain climate/soil-specific nut type WF-benchmarks	Addressing efficiency in the use of blue and green water resources. The average global blue plus green WF of e.g. unshelled almonds (6540 m^3^/ton) differs widely throughout geographical regions. Half of global almonds (with shell) are produced with a WF up to 4025 m^3^/ton (which can be set as benchmark), whereas 25% are produced with a WF up to 2390 m^3^/ton (Mekonnen and Hoekstra, 2014)(SI Table 3). When the WF is larger than the global benchmark, this means blue and green water resources are being used inefficiently. Bringing all almonds with a higher WF to the 50th percentile benchmark (4025 m^3^/ton), would reduce the total green-blue WF of almonds (10,328 million m^3^) with 48%. SI Table 3 shows different reduction potentials for all nuts. SDG indicator 6.4.1 deals with efficient water use. Different land and water management techniques as well as agricultural practices, as partly discussed by Willett et al. (2019), can achieve WF benchmarks, taking into account trade-offs with other environmental concerns and related planetary boundaries. Willett et al. (2019) did account for increased nutrient application to increase yields related to the planetary boundary biogeochemical flows, but not for a full environmental footprint family assessment which e.g. also includes chemical pollution from pesticides (Vanham et al., 2019a).
Choice where to produce nuts	The shifts in agricultural food type production, as proposed by Willett et al. (2019), provide opportunities to produce nuts on agricultural lands currently producing other commodities. Blue and green water resources on these lands should be used efficiently and sustainably.
Integrated Water Resources Management (IWRM), including the setting of river basin WF caps	IWRM, including through transboundary cooperation, and the establishment, management or optimisation of institutions to support it. SDG indicators 6.5.1 and 6.5.2 explicitly relate to IWRM. IWRM includes cross sectoral (e.g. food and energy security) and intra-sectoral (e.g. different agricultural commodities) water allocation as well as the maintenance of environmental flow requirements (Vanham et al., 2018b). The establishment of WF caps per river basin would add to achieve IWRM
Other solutions	Decrease of food loss and waste along the nut supply chain (FAO, 2013; Kummu et al., 2012; Willett et al., 2019)

Regarding blue WF unit amounts, especially pistachios are very water demanding both per unit of mass and protein. Groundnuts have generally smaller total and blue WFs both per kg and per gram of protein than tree nuts, but certain authors advocate to produce more perennial crops (treenuts) instead of annual crops (groundnuts) as they promise more sustainable agroecosystems (Crews et al., 2018).

## 4 Conclusions

Current global nut production contributes to and is affected by different levels of blue water stress, in many regions of the world. The results show for 7 different nut types combined, that 74% of all irrigated nuts are produced under blue water stress (moderate, significant or severe) and 63% under severe blue water stress. This was not considered in the recent study of Willett et al. (2019).

Latter study recommends for a substantial increase in global nut consumption, which requires a substantial increase in global nut production. To achieve such an increased production in a water-sustainable way, we discuss a list of essential options that need to be accounted for. Our study highlights that nuts are water-intensive agricultural commodities that require special attention in order to reach SDG 2 (food security) in harmony with SDG 6 (Ensure availability and sustainable management of water and sanitation for all).

## Declaration of competing interest

The authors declare that they have no known competing financial interests or personal relationships that could have appeared to influence the work reported in this paper.
