# Supplementary material for: Treenuts and groundnuts in the EAT-Lancet reference diet: Concerns regarding sustainable water use
Source: Glob Food Sec. 2020 Mar;24:100357. doi: 10.1016/j.gfs.2020.100357 (PMC7063698; doi:10.1016/j.gfs.2020.100357)
Supplement: Multimedia component 1 [file mmc1.docx]

**Supplementary Information (SI)**

Supplementary Table 1: Average protein content of groundnuts and treenuts. Source (FAO, 2019)

| **Food item** | **Protein content (g/kg)** |
| --- | --- |
| Groundnuts, shelled, | 257 |
| Cashew nuts, shelled* | 153 |
| Chestnuts, shelled or peeled | 18 |
| Almonds, shelled or peeled | 200 |
| Walnuts, shelled or peeled | 143 |
| Pistachios, whether or not shelled or peeled | 103 |
| Hazelnuts, shelled or peeled | 130 |

* calculated from cashew nuts using the production factor of 0.3 obtained from FAO (2003) Technical Conversion Factors

Supplementary Table 2: Production and water footprint of different nut types. Data sources: FAOSTAT (2019) and Mekonnen and Hoekstra (2011)

| **Nut type** | **Annual production (million tons/y)** | | | | | | | **Global WF (million m^3^/y)** | | | | |
| --- | --- | --- | --- | --- | --- | --- | --- | --- | --- | --- | --- | --- |
|  | **Total** | | | | **Irrigated** | | **Blue** | | | **Green** | | **Total** |
|  | **2000** | **2013** | **2017** | **1996-2005** | | | | | | | | |
| Groundnuts, with shell | 34.8 | 46.4 | 47.1 | 8.7 | | 5,107 | | | 84,149 | | 89,256 | |
| Groundnuts, shelled* | 24.4 | 32.5 | 33.0 | 6.1 | | 5,107 | | | 84,149 | | 89,256 | |
| Cashew nuts, with shell | 2.1 | 3.8 | 4 | 0.2 | | 1,845 | | | 25,749 | | 27,594 | |
| Cashew nuts, shelled** | 0.6 | 1.1 | 1.2 | 0.1 | | 1,845 | | | 25,749 | | 27,594 | |
| Chestnuts, whether or not shelled | 0.9 | 2.1 | 2.3 | 0.2 | | 171 | | | 2,396 | | 2,567 | |
| Almonds, with shell | 1.5 | 2.0 | 2.2 | 1.0 | | 3,013 | | | 7,315 | | 10,328 | |
| Almonds, shelled*** | 0.8 | 1.0 | 1.1 | 0.5 | | 3,013 | | | 7,315 | | 10,328 | |
| Walnuts, with shell | 1.3 | 3 | 3.8 | 0.7 | | 1,762 | | | 3,806 | | 5,568 | |
| Walnuts, shelled**** | 0.7 | 1.6 | 2.0 | 0.4 | | 1,762 | | | 3,806 | | 5,568 | |
| Pistachios, whether or not shelled | 0.6 | 0.7 | 1.1 | 0.4 | | 3,507 | | | 1,428 | | 4,935 | |
| Hazelnuts, with shell | 0.7 | 0.9 | 1 | 0.3 | | 798 | | | 2,794 | | 3,592 | |
| Hazelnuts, shelled***** | 0.4 | 0.5 | 0.5 | 0.2 | | 798 | | | 2,794 | | 3,592 | |
| Other nuts | 0.7 | 1.0 | 1.1 |  | | 842 | | | 12,841 | | 13,683 | |
| Tree nuts total, with shell | 7.8 | 13.5 | 15.5 | 2.8 | | 11,938 | | | 56,329 | | 68,267 | |
| Tree nuts total, shelled****** | 4.6 | 8.0 | 9.3 | 1.7 | | 11,938 | | | 56,329 | | 68,267 | |
| Groundnuts + treenuts, with shell | 42.6 | 59.9 | 62.6 | 11.5 | | 17,045 | | | 140,478 | | 157,523 | |
| Groundnuts + treenuts, shelled | 29.0 | 40.5 | 42.3 | 7.8 | | 17,045 | | | 140,478 | | 157,523 | |

* product fraction from with shell to shelled 0.7

**product fraction from with shell to shelled 0.3

*** product fraction from with shell to shelled 0.5

**** product fraction from with shell to shelled 0.53

***** product fraction from with shell to shelled 0.5

****** weighted average product fraction for treenuts based on 2017 production: 0.6

Supplementary Table 3: WF benchmarks of groundnuts and treenuts. Source Mekonnen and Hoekstra (2014)

| **Food item** | **Green-blue water footprint (m^3^/ton) at different production percentile** | | | | **Global average** |
| --- | --- | --- | --- | --- | --- |
|  | 10^th^ | 20^th^ | 25^th^ | 50^th^ |  |
| Almonds, with shell | 1,881 | 2,207 | 2,390 | 4,025 | 6,540 |
| Cashew nuts, with shell | 4,221 | 4,579 | 4,702 | 6,290 | 13,774 |
| Chestnut | 1,220 | 1,292 | 1,332 | 1,524 | 2,606 |
| Hazelnuts, with shell | 3,753 | 3,938 | 4,014 | 4,421 | 4,903 |
| Pistachios | 2,658 | 3,350 | 3,677 | 10,920 | 10,697 |
| Walnuts, with shell | 1,813 | 2,503 | 2,830 | 3,868 | 4,105 |
| Groundnuts in shell | 1,382 | 1,459 | 1,507 | 2,269 | 2,618 |
|  | **Global total green-blue water footprint (million m^3^/yr)** | **Potential green-blue water footprint reductions at different production percentile (%)** | | | |
|  |  | 10^th^ | 20^th^ | 25^th^ | 50^th^ |
| Almonds, with shell | 10,328 | 72 | 67 | 65 | 48 |
| Cashew nuts, with shell | 27,594 | 70 | 67 | 66 | 60 |
| Chestnut | 2,567 | 53 | 50 | 49 | 45 |
| Hazelnuts, with shell | 3,592 | 24 | 20 | 18 | 10 |
| Pistachios | 4,935 | 75 | 69 | 67 | 32 |
| Walnuts, with shell | 5,568 | 58 | 43 | 36 | 19 |
| Groundnuts in shell | 89,256 | 47 | 45 | 43 | 27 |

**Literature**

FAO. Technical conversion factors. http://www.fao.org/fileadmin/templates/ess/documents/methodology/tcf.pdf. Food and Agriculture Organization of the United Nations, Rome, Italy, 2003.

FAO. Nutritive factors, http://www.fao.org/fileadmin/templates/ess/ess_test_folder/Food_security/Excel_sheets/Nutritive_Factors.xls, 2019.

FAOSTAT. Food and agriculture data of the FAO, http://www.fao.org/faostat/en/, 2019.

Mekonnen MM, Hoekstra AY. The green, blue and grey water footprint of crops and derived crop products. Hydrol. Earth Syst. Sci. 2011; 15: 1577-1600 10.5194/hess-15-1577-2011

Mekonnen MM, Hoekstra AY. Water footprint benchmarks for crop production: A first global assessment. Ecological Indicators 2014; 46: 214-223 https://doi.org/10.1016/j.ecolind.2014.06.013
